# Cognitive Demand and Accommodative Microfluctuations

**DOI:** 10.3390/vision2030036

**Published:** 2018-09-06

**Authors:** Niall J. Hynes, Matthew P. Cufflin, Karen M. Hampson, Edward A. H. Mallen

**Affiliations:** 1 School of Optometry & Vision Science, University of Bradford, Bradford BD7 1DP, UK; 2 Department of Engineering Science, University of Oxford, Oxford OX1 3PJ, UK

**Keywords:** accommodation, micro-fluctuations, cognitive demand

## Abstract

Previous studies have shown cognition to have an influence on accommodation. Temporal variation in the accommodative response occurs during the fixation on a stationary target. This constantly shifting response has been called accommodative micro-fluctuations (AMFs). The aim of this study is to determine the effects of increasing task cognitive demand on the ocular accommodation response. AMFs for 12 myopes and 12 emmetropes were measured under three conditions of varying cognitive demand and comprising reading of numbers (Num), simple arithmetic (SA), and complex arithmetic (CA). Fast Fourier transforms were used to analyze the different frequency band components of the AMFs. Other aspects of AMFs including root mean square accommodation values and chaos analysis was applied. A repeated measures ANOVA revealed a significant main effect of cognition in the mean power of the high frequency component (HFC) (F_2,44_ = 10.03, *p* < 0.005). Pairwise analyses revealed that these differences exist between SA and CA tasks (*p* < 0.005) and the Num and CA (*p* < 0.005) tasks with the HFC power being the highest for the CA condition. It appears that the difficulty of a task does affect active accommodation but to a lesser extent than other factors affecting accommodation.

## 1. Introduction

An accurate ocular accommodation response is a requirement for pre-presbyopes to minimize retinal defocus and maintain clear viewing of a near-work task.

Temporal variation in the accommodative response occurs during the fixation on a stationary target and this constantly shifting response has been termed accommodative micro-fluctuations (AMFs) [1,2]. These AMFs are approximately 0.50 D in amplitude [3]. Fast Fourier Transforms (FFTs) have indicated that the AMFs consist of two main contributing frequencies bands. This includes a high frequency component (HFC, typically between 1.0 and 2.3 Hz) and a low frequency component (LFC, frequencies below 0.6 Hz) [1,3,4,5,6]. The origin of the HFC branch of the micro-fluctuations has been linked to the arterial pulse [4,7] while the LFC micro-fluctuations are thought to be derived from a neurological source [8]. LFC has been shown to increase under similar circumstances that cause the depth of focus to increase [3]. A positive correlation between an objective depth of focus and AMFs has been demonstrated, which suggests a possible role in blur sensitivity [9]. Many factors have been shown to influence AMFs including age [6,10,11,12], luminance [13,14], pupil diameter [1,5,13], refractive error [13,15], and accommodative demand [16,17,18]. It has been suggested that the LFC may have a role to play in fine accommodation control. However, there is still much uncertainty about this [3].

The cognitive context of any near-work task must also be considered since cognitive demand can influence steady-state accommodation [19,20,21,22,23,24,25,26,27,28]. The mean accommodative response has been shown to be significantly increased during active cognitive tasks under conditions of greater accommodative demand [19]. These previous studies have investigated the mean accommodative response only and there is a paucity of research investigating the influence of cognitive demand on the AMFs. A recent study has examined AMFs and a cognitive demand finding increased AMFs in passive cognition conditions compared to active conditions [29].

The accommodation response including AMFs has been suggested as one of the contributing factors for Digital Eye Strain (DES) [30]. AMFs have been shown to increase under certain conditions [5,13,14]. For instance, under increased accommodative demand, AMFs rise in numbers [16,17,18]. If cognitive demand is found to increase AMFs, then tasks of increasing cognitive demand may further exacerbate DES-related symptoms. Chaos analysis has been used to examine the data since it can be a more sensitive measure of changes in AMFs than traditional power spectrum methods [31]. This study investigated the effect that varying degrees of active cognitive demand has on the AMFs.

## 2. Materials and Methods

### 2.1. Participants

Twenty-four participants were recruited, which are comprised of equal numbers of emmetropes and myopes (details in Table 1). All participants were between the ages of 18 and 35. Myopes were defined as participants with a mean spherical equivalent (MSE) refractive error greater >−0.50 D while those with a MSE refractive error of between −0.50 D and +0.75 D were classed as emmetropic. The refractive error of myopic participants had been stable for at least one year prior to the experiment. Astigmatism did not exceed −1.00 D for all participants. All participants achieved +0.00 logMAR or better in their habitual correction on a Bailey-Lovie logMAR chart and were free of any ocular pathology. Participants were excluded if they had a lag of accommodation greater than 1.50 D when viewing a number target subtending a visual angle of 0.86 degrees at 33 cm binocularly. Participants gave their consent to take part in the experiment. The study was approved by the Biomedical, Natural, and Physical Sciences Research Ethics Panel at the University of Bradford and this study was undertaken in accordance with the Declaration of Helsinki.

Soft contact lens correction was used for all myopes to equalize accommodative demand across all participants. The majority of participants (11) wore their own contact lenses and the residual refractive error was corrected to ensure that it was ≤0.25 DS. In cases where the participant was not a habitual contact lens wearer, they were fitted by with daily disposable contact lenses (1-Day Acuvue Moist, Johnson and Johnson) and were allowed 20 min to adapt. Soft contact lenses have previously been found to have no influence on AMFs [32].

Amplitude of accommodation was measured using an RAF rule to ensure that participants could exert at least 6 D of accommodation, which is double the accommodative demand required for the task [33]. This was done to establish that the amplitude of accommodation was clinically normal.

### 2.2. Instrumentation

The accommodative response was monitored using a modified Shin-Nippon SRW-5000 autorefractor, which allowed for the continuous recording of accommodation at a sampling rate of 22 Hz [34,35]. This autorefractor has been validated in children [36] and adults [34] in its unmodified form and has been used in previous research investigating AMFs [13,15,16,21,28]. Pupil size was monitored on a built-in screen, which ensures that the pupil diameter remained above 2.9 mm throughout data collection. Participant’s viewed the targets binocularly while the accommodative response was recorded monocularly. The participant’s right eye was used in all cases with the exception of participant LG whose left eye response was recorded due to it having less astigmatism than the right eye.

Each participant’s head movement was restricted using a modified headband rest that secured the top of the participants head and the participants were required to keep their forehead pressed against the headrest. A chin rest or bite-bar was unsuitable since the participants were required to give verbal feedback during the experiment, which would have led to excessive and disruptive head movement. Verbal feedback was requested from the participants in response to the cognitive tasks, which are detailed below.

The high contrast fixation targets for the various tasks were displayed on a 17′′ LCD monitor (MultiSync LCD175VXM+, NEC Corporation, Tokyo, Japan). The luminance was measured at 23.9 cd/m^2^.

### 2.3. Tasks

The tasks took place in a room illuminated by a LED tube lighting measuring 880 lux.

Initially, each participant undertook a baseline measure of accommodation while viewing a Maltese cross target at a distance of 6 m. The luminance of this target was 68.9 cd/m^2^.

Three tasks vary in active cognitive demand, which were performed in a randomized order by participants, and were as follows.

- Task (1) observation of single and two digit randomized numbers with a requirement to read them aloud. They appeared on the computer monitor (Num).
- Task (2) addition or subtraction of a random selection of single-digit numbers when they appeared on the computer monitor to give a verbal answer (SA).
- Task (3) addition or subtraction of a random selection of double-digit numbers when they appeared on the computer monitor to give a verbal answer (CA).

Numbers appeared in the same part of the screen, which allowed the participant to use them as a fixation point from a distance of 0.33 m/accommodative demand of 3 dioptres (D). The visual angle subtended for the Num, SA, and CA tasks was horizontally 0.86, 1.38, and 2.08 degrees, respectively, and 0.86 degrees vertically. Each task lasted up to one minute to ensure there was 20 s of usable data. The participant was given a break of approximately one minute in between each task. Participants were instructed to keep the accommodative targets as clear as possible throughout the tasks. They were asked to give verbal feedback indicating which number was on the screen (task 1) or the sum of two numbers (task 2 or 3). The numbers presented were changed immediately following an answer by the participant. The change in cognitive demand was similar to that used in other accommodation studies [20,24].

## 3. Analysis

Two accommodation traces (each 20 s in duration) were recorded from each participant and for each of the three tasks. The two measures per participant were averaged. Blinks were removed from the data in an Excel program by identifying accommodative response measures that were 2 D away from the accommodative demand and replaced with an average of the five previous data points before the blink when measuring the mean accommodative response [31,37]. When AMFs were analyzed using Matlab (Matlab R2013a, The Mathworks, Inc., Natick, MA, USA), blinks were removed from the data by identifying accommodative response measures that were 2 D away from the accommodative demand and replacing them using interpolation based on the research conducted by Hampson and colleagues [38]. 

Data analysis was carried out on the different conditions using repeated measure ANOVAs through SPSS (IBM Corp., Released 2013. IBM SPSS Statistics for Windows, Version 22.0. Armonk, NY, USA, IBM Corp). Results were deemed statistically significant when *p*-values of <0.05 were present. Data related to the LFC, mid-frequency component (MFC) and HFC was derived by Fast Fourier Transform functions in Matlab. The LFC was measured between 0 and 0.6 Hz, the MFC between 0.7 and 0.9 Hz, and the HFC between 1.0 and 2.1 Hz. Root mean square (RMS) values were calculated and contrasted using this software.

Chaos Analysis was also used to determine if there was a significant difference between conditions. Healthy physiological signals such as the heartbeat display chaos [39]. Unlike the traditional definition of chaos, chaotic systems have underlying laws that describe their behavior. A feature of chaotic systems is their sensitivity to the initial conditions. This means that a minute change in a variable can cause the system to behave very differently with this difference increasing over time. This can be measured using the Lyapunov exponent. The larger the value of the exponent, the greater the sensitivity to the initial conditions. A decrease in the Lyapunov exponent can indicate stress to the system or ill-health [40].The largest Lyapunov exponent (LLE) value was calculated for each condition to determine if there was a change in the amount of chaos in the accommodative system dependent on the condition [31,38]. A reduction in the Lyapunov exponent for a given 20-second accommodative trace would indicate that the accommodative system is under stress.

## 4. Results

### 4.1. Mean Accommodative Response

**Figure 1:**
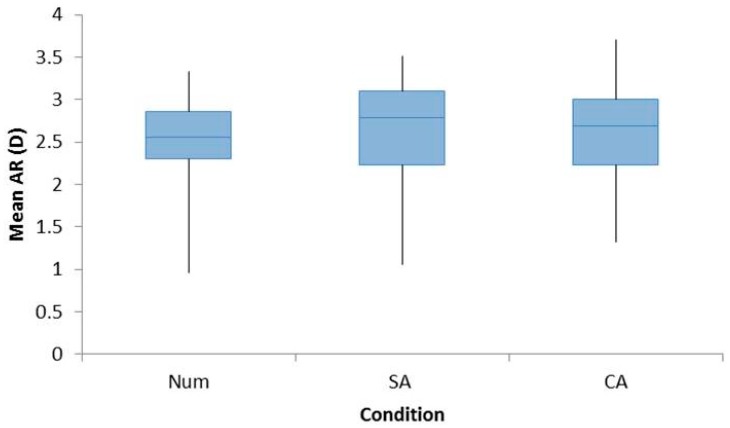
Box plots demonstrating the increase in the accommodative response from baseline distance readings for all participants for the conditions Num, SA, and CA. A repeated measures ANOVA with Bonferroni post-hoc analysis found no significant differences across the three conditions.

The Refractive Error group was found to have no significant effect on the mean accommodative response. Hence, the data for both groups were combined into a single group for analyses (F_1,22_ = 0.606, *p* = 0.445). The mean accommodative response was found to be unaffected by cognitive demand with values equivalent across the three tasks (Num: 2.53 ± 0.55 D, SA: 2.58 ± 0.69 D, and CA: 2.58 ± 0.55 D, F_2,44_ = 0.454, *p* = 0.638), which is shown in Figure 1.

### 4.2. RMS Accommodation

**Figure 2:**
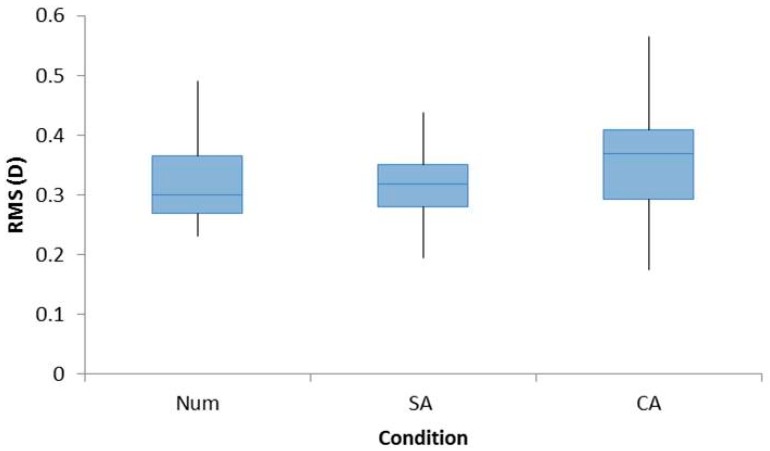
Box plots demonstrating the RMS accommodation for all participants for the conditions Num, SA, and CA. A repeated measures ANOVA with Bonferroni post-hoc analysis found a significant main effect, but no significant effect was found for pair-wise analyses.

There was no significant effect of a refractive error group for RMS (F_1,22_ = 1.123, *p* = 0.301) so data were pooled into a single group. A significant effect of cognition was observed (F_2,44_ = 4.030, *p* < 0.05) with a higher trend for the CA condition observed. However, there was no significant effect when pairwise comparisons were analyzed between Num and SA (*p* = 1.000), Num and CA (*p* = 0.075), and SA and CA (*p* = 0.059). The RMS values are shown in Figure 2.

### 4.3. Fast Fourier Transform

The accommodative traces underwent FFT to isolate the low (LFC), medium (MFC), and high (HFC) frequency components of the signal. The effect of cognition on each of these components was examined. There were no significant differences found between myopes and emmetropes for LFC (F_1,22_ = 1.197, *p* = 0.286), MFC (F_1,22_ = 0.036, *p* = 0.851), or HFC (F_1,22_ = 0.150, *p* = 0.702). Therefore, the data was pooled.

**Figure 3:**
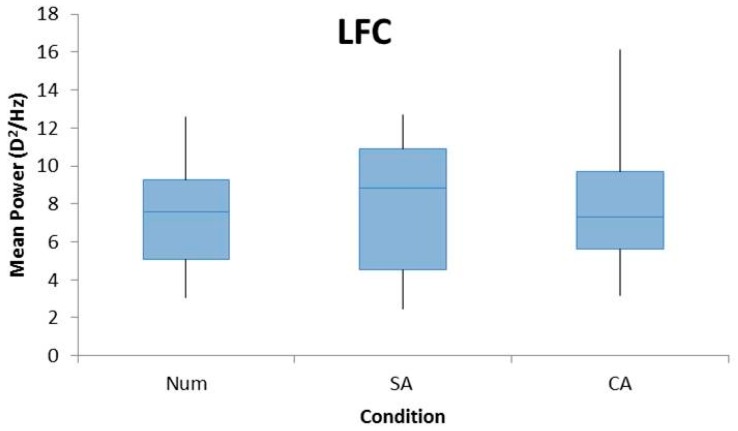

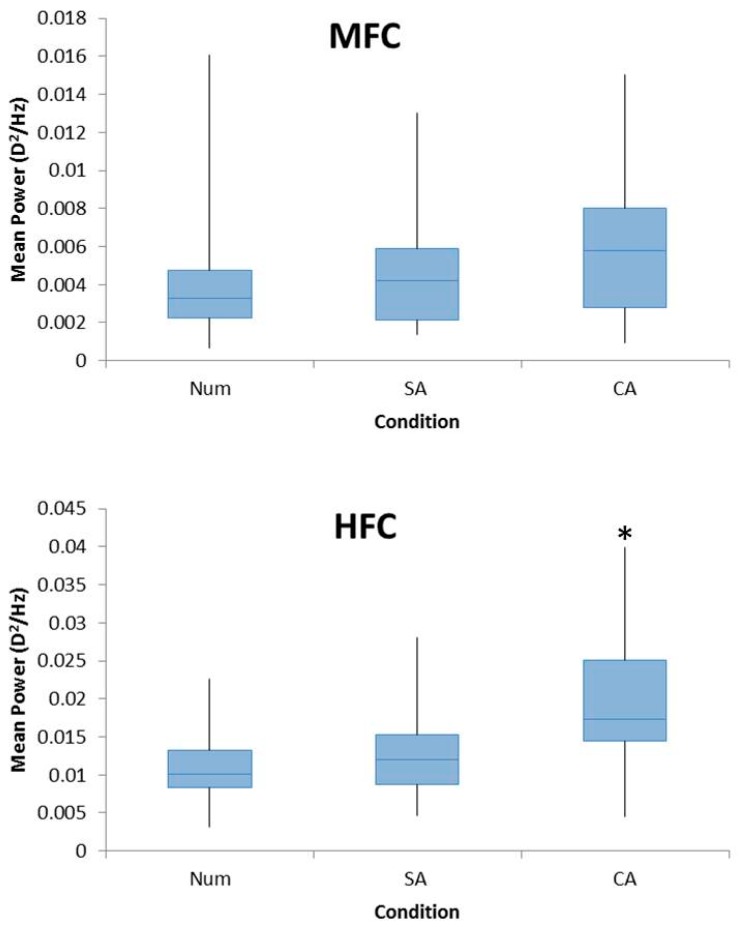
Box plots demonstrating the mean power of FFTs for the LFC (**top**), MFC (**middle**), and HFC (**bottom**) for all participants for the conditions Num, SA, and CA. A repeated measures ANOVA with Bonferroni post-hoc analysis found a significant effect for HFC with a significant difference between CA and SA and CA and Num conditions. No significant effect was found for LFC or MFC.

A repeated measures ANOVA revealed a significant main effect of cognition in the mean power of the HFC (F_2,44_ = 10.03, *p* < 0.005). Pairwise analyses revealed that these differences exist between SA and CA tasks (*p* < 0.005), and the Num and CA (*p* < 0.005) tasks with the HFC power being the highest for the CA condition, which is shown in Figure 3. There was no significant difference found between the Num and SA conditions (*p* = 0.965).

There was no significant effect of cognition on the LFC (F_2,44_ = 0.821, *p* = 0.447) or MFC (F_2,44_ = 2.513, *p* = 0.093) among the three conditions (Figure 3).

### 4.4. Chaos Analysis

A refractive error was found to have no effect on LLE (F_1,22_ = 1.743, *p* = 0.200). Cognition was found to have no effect on LLE values with equivalent values found for all three cognitive conditions (F_2,44_ = 0.070, *p* = 0.933).

## 5. Discussion

The influence of cognitive demand on AMFs was investigated in this study with AMFs measured in a group of young adult participants under three different cognitive demands. A significant increase in the power of the HFC was observed when participants undertook the task with the highest cognitive demand. In addition, cognition was also found to affect RMS accommodation readings for the three conditions. We found no significant changes in the mean accommodative response or in the power of the LFC, MFC, or chaos analysis in the AMFs when cognitive demand was varied. We hypothesize that there may be a number of reasons for this.

### 5.1. Mean Accommodative Response

There was no significant difference noted in the mean accommodative response across the three conditions. Kruger [24] found an increase in the accommodative response using similar tasks to this experiment. A major methodological difference between our study and Kruger’s study is that all tasks were completed by the same participants in this experiment. In Kruger’s study, there was a separate control group for reading numbers, which was compared to the experimental group that were adding and subtracting numbers. Other studies have reported no significant difference in the accommodative response across varying cognitive demands [20,23,29]. Roberts and colleagues [29] found no difference in the lag of accommodation in adults but did note an increased lag in children for the passive condition. Bullimore and Gilmartin [20] found no significant difference between passive and active conditions for an accommodative demands of 1 D and 3 D. A significant shift in accommodation for the same experiment at an accommodative demand of 5 D was noted. It could be hypothesized, therefore, that age and accommodative demand have stronger contributory influences when cognitive demand and accommodation is assessed. Jainta and colleagues [23] proposed gaze changes as a possible reason why the accommodative response may change as opposed to it being cognitively induced. Previous studies have investigated the influence of gaze eccentricity on the accommodative measures recorded by the Shin-Nippon SRW-5000 autorefractor used in this experiment [41] and found that the measures were relatively unaffected up to eccentricities of 10 degrees. As the maximum change in eccentricity from the center of a fixation target to the edge of the target was less than 1.25 degrees, it is unlikely that this would have affected accommodation measurements during the current experiment.

### 5.2. Factors Affecting AMFs and Accommodation

There are a number of factors that can lead to a change in the nature of AMFs. Gray and colleagues [14] and Day and colleagues [13] investigated the effect that the pupil diameter had on AMFs by finding an increase in the LFC of AMFs when the pupil diameter decreased below 2 mm. The pupil diameter appeared to have little effect on the HFC in these studies. The Shin-Nippon SRW-5000 autorefractor requires a pupil diameter greater than 2.9 mm [41] to allow an accurate recording of dynamic accommodation. As a result, participants with pupils smaller than 2.9 mm were excluded from the study and, therefore, it would be unlikely that the pupil diameter had an effect on the AMFs in this experiment. Accommodative demand [16,17,18] and luminance [13,14] factors were kept constant to prevent an effect they may have on the experiment.

### 5.3. Refractive Error and AMFs

Previous studies have noted a difference in AMFs between myopes and emmetropes [13,15,16]. In this study, we have found a trend for higher values in myopes, but this was not statistically significant. This is in contrast to the results found by Day and colleagues [16] in which they found an increase in the RMS values for myopic participants compared to emmetropes at an accommodative demand of 3 D. Similar accommodative responses between emmetropes and early-onset myopes have been noted in an experiment examining retinotopic accommodation responses in progressive myopia [42]. Emmetropes and early-onset myopes demonstrated significantly smaller AMFs than late-onset myopes. Early-onset myopes formed the majority of this current experiment, which may be an explanation for the lack of a significant effect found between refractive groups. This could be addressed by including equal groups of emmetropes including early-onset and late-onset.

### 5.4. Recent Research on Cognitive Demand and AMFs

Roberts and colleagues [29] investigated the effects of cognitive demand on AMFs in children and adults for passive and active cognitive conditions. They found that RMS accommodation and LFC values were higher for the passive condition than the active condition in children. A reduced difference was found in adults, which was significant for the LFC but not significant for RMS accommodation. There are a number of differences to recognize between this study and ours. All participants in our study were adults and were either emmetropic or myopic while Roberts and colleagues had predominantly children ranging from emmetropia to hyperopia in their study. We compared three active cognitive conditions while they investigated the difference between passive and active conditions. However, their findings do point to the LFC potentially being more changeable between passive and active conditions than over different levels of active cognition.

### 5.5. Changes in HFC

Cognitive demand has been suggested to cause an increase in heart rate in previous studies [43,44]. It has been proposed that this increase in the heart rate during cognitive processing is to allow increased metabolic activity in the brain [44]. In this experiment, a significant increase in the power of the HFC is apparent with increasing cognitive demand. It may be that this is as a result of the arterial pulse increasing during the more difficult parts of the cognitive tasks. Winn and colleagues [4] previously observed an association between arterial pulse and the HFC in AMFs. The LFC appears not be affected by cognition in this experiment, which suggests that cognition appears to have a stronger effect on the HFC rather than the LFC.

Further examination into this could involve conducting a similar experiment while simultaneously monitoring a participant’s heart rate to detect if there is any change among conditions. Previous research looked at the effect that varying cognitive demand had on the mean accommodative response and cardiovascular function in different refractive groups [21]. A piezoelectric pulse transducer was used to record cardiovascular function. A similar system while monitoring AMFs could potentially be used to determine if there is a correlation between any change in AMFs and cardiovascular function.

### 5.6. RMS Accommodation

The cognitive demand of the task produced a significant main effect on RMS accommodation values with the CA task displaying higher values than the Num and SA tasks. However, there was not a significant effect in pairwise analyses as seen with the HFC. Recent research examining cognitive demand and AMFs found no significant difference in RMS accommodation values between passive and active cognitive conditions in adults [29]. However, a significant shift in AMFs was found in children during passive viewing conditions, which may infer that any effect cognitive demand may potentially have on AMFs is limited by age. This suggests that the influence of the cognitive demand is limited in adults when compared to other factors such as target proximity [3,16,17,18] or pupil diameter [1,3,5,13]. Day and colleagues [16] demonstrated an increase in both RMS accommodation and LFC values when the accommodative demand increased from 0 D to 4 D. A similar result is seen in a study into micro-fluctuations and luminance where the LFC and RMS accommodation values are both relatively higher in lower luminance conditions [14]. In both of these experiments, there appears to be no significant effect on the HFC. In our experiment, no significant change was found in the LFC while there was for the HFC. Taking this into account with an uncertain RMS accommodative response, it could be suggested that the changes observed in the AMFs are not down to an active neurological change. It is more likely that the changes are due to other factors occurring elsewhere in the system. 

### 5.7. Inter-Participant Variation

A limitation to this experiment was high inter-participant variability, which is a phenomenon that has been documented across many studies related to AMFs [3,13,19,24,45,46,47]. Suggestions for this include that some participants may have found the experiment more stressful than others [19] or that higher order aberrations may possibly have a role to play [45]. There are cases in experiments where some participating AMFs despite having a normal accommodative response simply do not respond to the stimulus and have been removed from the study [3]. Similar variability was also observed in this study. This could be attributable to participant anxiety. For instance, participants worried about the difficult arithmetic when compared to the more simple task of reading numbers. The variability was more noticeable in the LFC with some participants demonstrating a much higher power than others.

Day and colleagues [14] reported individual variability during their investigation into the influence of the depth of focus on AMFs in myopes and emmetropes. They found that there was no correlation between the high inter-subject variability and the size of the effect ocular depth of focus had on AMFs. They concluded that the variation may be due to factors of individual anatomy or innervation.

### 5.8. Chaos Theory Analysis

Previous research has suggested that AMFs may have an input in chaotic dynamics within the accommodative system [38]. In their experiment, Hampson and Mallen examined the defocus and spherical aberration Zernike coefficients. They found an increased LLE value in the defocus condition. However, due to high variability between subjects, this was not statistically significant. A similar high variability was noted in this experiment.

A similar trend to a later paper that measured LLE values in myopes and emmetropes was found in this study. Higher LLE values were observed in emmetropes when compared to myopes. This is significant when emmetropes were compared to late-onset myopes [31]. Our findings were not statistically significant. However, this may be due to a majority of early onset myopes in that myopic group.

## 6. Conclusions

A significant effect was noted in the HFC of accommodative micro-fluctuations for more difficult levels of cognitive demand. The same effect, albeit reduced, was seen in the RMS values. Given these results, it appears that the difficulty of a task does affect active accommodation but to a lesser extent than accommodative demand or target luminance would.

## Figures and Tables

**Table 1:** Information regarding participants in the experiment. ± values refer to one standard deviation of the mean.

Refractive Group	Emmetropes	Myopes
No. of participants	12	12
Mean Age (yrs)	27.77 ± 4.13	24.15 ± 4.47
Range (yrs)	21–35	20–31
Mean Refractive Error (D)	+0.35 ± 0.20	−3.31 ± 2.10
Range of Mean Sph (D)	0.00 to +0.75	−1.00 to −6.88
Range of Amplitude of Accommodation	6.00–10.00 D	6.00–13.00 D
